# Disruption of the astrocyte–neuron interaction is responsible for the impairments in learning and memory in 5XFAD mice: an Alzheimer’s disease animal model

**DOI:** 10.1186/s13041-021-00823-5

**Published:** 2021-07-10

**Authors:** Moonseok Choi, Sang-Min Lee, Dongsoo Kim, Heh-In Im, Hye-Sun Kim, Yun Ha Jeong

**Affiliations:** 1 Department of Neurodegenerative Diseases Research Group, Korea Brain Research Institute (KBRI), 61, Cheomdan-ro, Dong-gu, Daegu, 41062 Republic of Korea; 2 Center for Neuroscience, Brain Science Institute, Korea Institute of Science and Technology (KIST), 5 Hwarang-ro 14-gil, Seongbuk-gu, Seoul, 02792 Republic of Korea; 3 Department of Pharmacology and Biomedical Sciences, College of Medicine, Seoul National University, 103 Daehakro, Jongro-gu, Seoul, 03080 Republic of Korea; 4 Seoul National University College of Medicine, Bundang Hospital, 82, Gumi-ro 173beon-gil, Bundang-Gu, Sungnam, 13620 Republic of Korea; 5 Neuroscience Research Institute, College of Medicine, Seoul National University, 103 Daehakro, Jongro-gu, Seoul, 03080 Republic of Korea

**Keywords:** Astrocyte–neuron interaction, Learning impairments, Memory impairments, Alzheimer’s disease, 5XFAD mice

## Abstract

The morphological dynamics of astrocytes are altered in the hippocampus during memory induction. Astrocyte–neuron interactions on synapses are called tripartite synapses. These control the synaptic function in the central nervous system. Astrocytes are activated in a reactive state by STAT3 phosphorylation in 5XFAD mice, an Alzheimer’s disease (AD) animal model. However, changes in astrocyte–neuron interactions in reactive or resting-state astrocytes during memory induction remain to be defined. Here, we investigated the time-dependent changes in astrocyte morphology and the number of astrocyte–neuron interactions in the hippocampus over the course of long-term memory formation in 5XFAD mice. Hippocampal-dependent long-term memory was induced using a contextual fear conditioning test in 5XFAD mice. The number of astrocytic processes increased in both wild-type and 5XFAD mice during memory formation. To assess astrocyte–neuron interactions in the hippocampal dentate gyrus, we counted the colocalization of glial fibrillary acidic protein and postsynaptic density protein 95 via immunofluorescence. Both groups revealed an increase in astrocyte–neuron interactions after memory induction. At 24 h after memory formation, the number of tripartite synapses returned to baseline levels in both groups. However, the total number of astrocyte–neuron interactions was significantly decreased in 5XFAD mice. Administration of Stattic, a STAT3 phosphorylation inhibitor, rescued the number of astrocyte–neuron interactions in 5XFAD mice. In conclusion, we suggest that a decreased number of astrocyte–neuron interactions may underlie memory impairment in the early stages of AD.

**Supplementary Information:**

The online version contains supplementary material available at 10.1186/s13041-021-00823-5.

## Introduction

The bidirectional communication between neurons and astrocytes on synapses is called a tripartite synapse [1]. Astrocytes uptake neurotransmitters to eliminate excitotoxicity at the synapse. Astrocytes also release gliotransmitters, such as glutamate, D-serine, GABA, and ATP, at the synapse. These gliotransmitters bind to neuronal receptors such as N-methyl-D-aspartate receptors (NMDARs) to modulate neuronal firing [2].

Alzheimer’s disease (AD) is characterized by amyloid plaques and neurofibrillary tangles in the brain [3]. AD is also associated with neuronal loss and gliosis, including those of reactive astrocytes [4], which are found in neurodegenerative diseases such as AD [5]. Reactive astrocytes have specific morphological characteristics, such as increased cell volume, dendritic thickness, and number of processes [6].

Our previous study had reported that astrocytes are activated in the reactive state via the JAK/STAT3 pathway in 6-month-old 5XFAD mice [7]. Astrocytes showed dynamic changes in the number of processes during memory induction with contextual fear conditioning [8]. The administration of a STAT3 phosphorylation inhibitor, Stattic, restored the cognitive function and astrocyte condition [7]. However, the mechanisms by which reactive astrocytes affect astrocyte–neuron interactions during memory formation remain to be elucidated.

In this study, we assessed time-dependent morphological changes in astrocytes during hippocampal long-term memory formation in 6-month-old 5XFAD mice. In addition, we analyzed changes in astrocyte–neuron interactions in the hippocampal dentate gyrus (DG) during memory formation and after the administration of Stattic in 6-month-old 5XFAD mice.

## Results

In previous studies, we classified the type of astrocytes by morphological characteristic. Type II astrocytes appear a bipolar morphology and Type III astrocytes appear a radial morphology [8]. In this study, we focus on a morphological dynamics and astrocyte-neuron interaction during memory formation with the Type III astrocytes at DG in hippocampus.

**Fig. 1:**
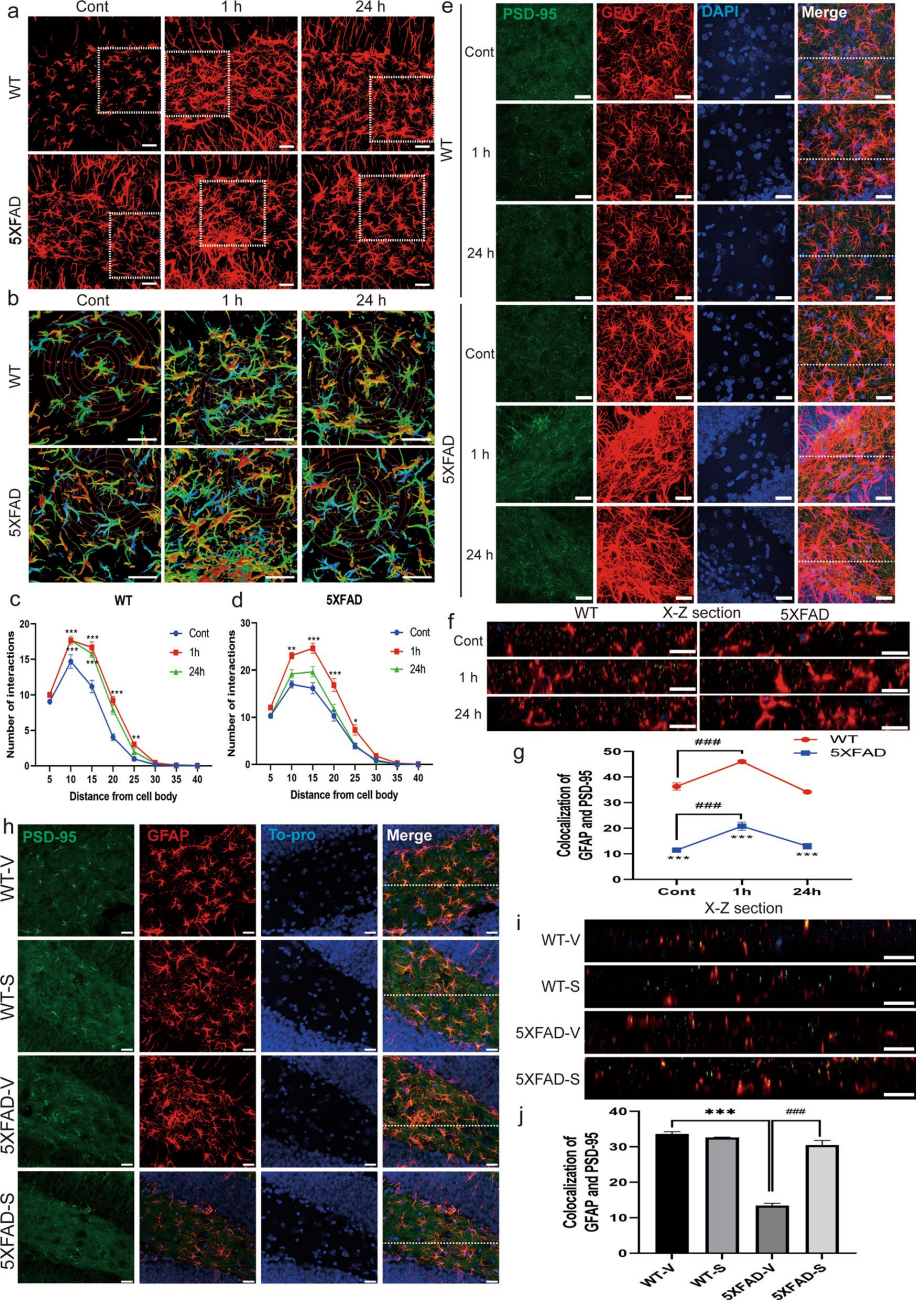
Astrocytes’ morphological dynamics and astrocyte–neuron interaction changes during memory formation in the hippocampal DG. **a** GFAP immunofluorescence in WT and 5XFAD mice after CFC; Z-projection depth is 0–30 μm (scale bar = 50 μm). **b** Gradation from red to blue in the 3D-reconstructed image; 2 × digital zoom from the 400 × original image, concentric circles are spaced at 10 μm (scale bar = 50 μm). **c** Quantification of the number of intersections between processes and each circle in the hippocampal DG; WT—Cont (N = 6), 1-h (N = 6), and 24-h (N = 6) groups. **d** Quantification of the number of intersections between processes and each circle in the hippocampal DG; 5XFAD—Cont (N = 9), 1-h (N = 10), and 24-h (N = 10) groups. **e** Immunofluorescence for PSD-95 and GFAP in hippocampal DG brain slices of WT and 5XFAD mice; Cont, 1-h, and 24-h groups (scale bar = 20 μm). **f** Reconstructed images showing a cross-section along the X–Z axis from a confocal Z-stack image from a WT and 5XFAD mouse; Cont, 1-h, and 24-h groups (scale bar = 20 μm). **g** Quantification of the colocalization of PSD-95 and GFAP in reconstructed cross-section images in the hippocampal DG; WT — Cont (N = 3), 1-h (N = 3), and 24-h (N = 3), 5XFAD—Cont (N = 3), 1-h (N = 3), and 24-h (N = 3) groups. **h** Immunofluorescence for PSD-95 and GFAP in the hippocampal DG of WT and 5XFAD mice that were treated with vehicle or Stattic (scale bar = 10 μm). **i** Reconstructed images showing a cross-section along the X–Z axis from a confocal Z-stack image from WT and 5XFAD mice that were treated with vehicle or Stattic (scale bar = 10 μm). **j** Quantification of the colocalization of PSD-95 and GFAP in the reconstructed cross-section images the hippocampal DG; WT-V (N = 4), WT-S (N = 4), 5XFAD-V (N = 4), 5XFAD-S (N = 4) groups. *p < 0.05, **p < 0.01, ***p < 0.001, one-way ANOVA with Fisher’s LSD post hoc analysis. Data are presented as mean ± SEM

To assess the morphological dynamics of Type III astrocytes with memory impairment, we performed Sholl analysis in 6-month-old 5XFAD mice after memory induction using contextual fear conditioning test (CFC). The number of astrocytic processes increased after 1 h and 24 h of memory induction in WT mice (Fig. 1a–c). These morphological astrocyte dynamics increased after 1 h only in 5XFAD mice, although the total number of processes was higher in 5XFAD mice (Fig. 1d).

5XFAD mice had a significantly decreased number of astrocyte–neuron interactions compared to WT mice (Fig. 1e–g), although the total number of astrocytes was increased compared to WT mice (Fig. 1d). During memory formation, the colocalization of PSD-95 and GFAP was significantly increased after 1 h and returned to the baseline level in 24 h in WT mice. Interestingly, in 5XFAD mice, the interaction between PSD-95 and GFAP was also increased at 1 h and returned to the baseline level at 24 h (Fig. 1g).

The colocalization of PSD-95 and GFAP was significantly decreased in the 5XFAD mice injected with vehicle (5XFAD-V) compared to the WT mice injected with vehicle (WT-V) (Fig. 1h, i). In addition, Stattic treatment increased the number of PSD-95 and GFAP colocalization in 5XFAD mice compared to that in 5XFAD-V mice (Fig. 1j). However, the protein expression level of GFAP was significantly decreased in 5XFAD mice injected with Stattic (5XFAD-S) than 5XFAD-V group whereas the expression of PSD-95 was not altered (Additional file 2: Fig. S1).

## Discussion

The novel functions of astrocytes in memory formation have been extensively studied in recent decades. The binding of neurotransmitters to the receptors in astrocytes does not generate an action potential but rather increases the intracellular calcium concentrations, causing gliotransmitter release in a calcium-dependent manner [9]. The released gliotransmitters can bind to pre- or postsynaptic receptors to regulate neuronal excitability and synaptic transmission. Glutamate released from astrocytes binds to presynaptic NMDAR to promote neurotransmitter release, while ATP released from astrocytes suppresses synaptic transmission [10].

Recently, reactive astrocytes were significantly increased in a hippocampal DG region in an Alzheimer’s disease model [11]. Reactive astrocytes display dynamic morphological changes, such as an increase in the number of processes [12]. In our previous study, we reported that the number of astrocyte processes increased in the DG during hippocampus-dependent long-term memory formation via CFC [8].

In this study, we assessed the morphological dynamics of astrocytes in the hippocampal DG of 6-month-old 5XFAD mice. Additionally, we examined the changes in astrocyte–neuron interaction in tripartite synapses during memory formation in 5XFAD mice and found that decreased colocalization between astrocytes and neurons was recovered through the inhibition of STAT3 phosphorylation in 5XFAD mice.

Recent studies of tripartite synapses in the central nervous system have focused on membrane channels or transporter proteins in astrocytes. The neurotransmitter–gliotransmitter negative feedback system has been found to be altered at the tripartite synapse because of cognitive impairment in a schizophrenia mouse model [13]. Additionally, the glutamate-glutamine shuttling system via glutamate uptake transporters was deregulated at the tripartite synapse in AD-related cognitive impairment [14]. In our study, we found a similar pattern of changes in the astrocyte–neuron interaction in the hippocampal DG during memory formation between WT and 5XFAD mice. We also revealed a decreased colocalization between astrocytes and neurons in 5XFAD mice, which might be the reason for memory impairment in the CFC test. These results suggest that memory induction stimulates an increase in astrocyte–neuron interaction during memory formation and that a sufficient number of astrocyte–neuron interactions would be required for long-term memory formation in the hippocampal DG.

In conclusion, the decreased number of astrocyte–neuron interactions during the reactive state of astrocytes may partially underlie cognitive impairment in early-stage AD. Furthermore, a drug that specifically inhibits STAT3 phosphorylation in astrocytes may represent a novel therapeutic strategy for early-stage AD.

In future studies, we will focus on the interaction of molecular and morphological changes between neurons, astrocytes, and microglia in the hippocampus during memory processes such as long-term memory formation, consolidation, and retrieval.

## Supplementary Information

**Additional file 1.** The experiment methods including the statistical analysis details are provided as a additional file.

**Additional file 2.** Additional figure for the change of protein expression of GFAP and PSD-95 after Stattic administration in the hippocampus.

## Data Availability

Detailed materials and methods are included in Additional file 1. All data supporting the finding of this study are available from the corresponding author on reasonable request.

## Abbreviations

AD: Alzheimer’s disease
CFC: Contextual fear conditioning
DG: Dentate gyrus
GFAP: Glial fibrillary acidic protein
NMDARs: N-methyl-D-aspartate receptors
PBS: Phosphate-buffered saline
PDS-95: Postsynaptic density protein 95
SEM: Standard error of the mean
