# Supplementary material for: Disruption of the astrocyte–neuron interaction is responsible for the impairments in learning and memory in 5XFAD mice: an Alzheimer’s disease animal model
Source: Mol Brain. 2021 Jul 10;14:111. doi: 10.1186/s13041-021-00823-5 (PMC8272251; doi:10.1186/s13041-021-00823-5)
Supplement: Supplementary file 1 — Additional file 1. The experiment methods including the statistical analysis details are provided as a additional file. [file 13041_2021_823_MOESM1_ESM.docx]

**Disruption of the astrocyte–neuron interaction is responsible for the impairments in learning and memory in 5XFAD mice: an Alzheimer’s disease animal model**

Moonseok Choi, Sang-Min Lee, Dongsoo Kim, Heh-In Im, Hye-Sun Kim*, and Yun Ha Jeong*

**Methods and Materials**

***Reagents and antibodies***

Anti-GFAP antibody (#Z0334) was purchased from DAKO (Carpinteria, CA). Anti- PSD-95 antibodies (#MA1-046), anti-rabbit tagged Alexa Fluor 488 (#A11034) antibodies, anti-mouse tagged Alexa Fluor 555 (#A21426) antibodies, and 4’,6-diamidino-2-phenylinodole (#D3571) were purchased from Thermo Fisher (Waltham, MA).

***Experimental animals***

The Alzheimer’s disease model 5XFAD mice were purchased from Jackson Laboratories (strain: B6SJL-Tg [APP Sw, Fl, Lon, PS1, M146L, L286V] 6799Vas/J; Bar Harbor, ME) and maintained by crossing hemizygous transgenic mice with B6SJL F1 mice. Six-month-old 5XFAD mice were used in the experiments. The mice were housed per cage with a 12-h light/dark cycle and ad libitum access to food and water under standard laboratory housing conditions.

**Oral administration of Stattic**

Six-month-old WT and 5XFAD mice were freely administrated Sttatic (25 mg/kg per day) dissolved in water. Control groups as WT-vehicle and 5XFAD-vehicle were freely administrated only water. All groups of mice were scarified after 15 days’ treatment.

***Contextual fear conditioning***

Contextual fear conditioning was performed as previously described [[1]](#_ENREF_1). Briefly, on the first day, all experimental mice were habituated in the chamber for 5 min. On the second day, each mouse underwent the training session in the habituated chamber (13 × 13 × 25 cm) for 3 min with three repetitions of a foot-shock (0.7 mA, 2 s) at 1-min intertrial intervals. The 1-h group was sacrificed at 1 h after the training session. The 24-h group was sacrificed at 24 h after the training session.

***Immunofluorescence***

Immunofluorescence was performed as previously described [[2]](#_ENREF_1). Briefly, brains were fixed with 4% paraformaldehyde. The fixed brain tissue was sectioned into 30-μm thick slices using a cryotome. For antigen retrieval, the brain sections were heated in a 85°C water bath with 10 mM citrate acid (pH 6.0). The blocking solution was contained in 2% BSA and 0.3% Triton X-100 in PBS. Primary anti-GFAP (1:1,000) PSD-95 (1:100) and synaptophysin (1:1,000) antibodies in blocking solution were applied for 16 h at 4°C. Secondary anti-rabbit-488 (1:200) and anti-mouse-555 (1:200) antibodies in blocking solution were applied for 2 h at 24°C. For counter staining, the stained brain sections were incubated with 1 uM To-pro in PBS for 10 min at 24°C or DAPI contained mounting solution. The stained brain sections were imaged by confocal microscopy using LSM 510 (Carl Zeiss, Oberkochen, Germany) and Confocal-A1 (Nikon Corporation, Shinagawa, Japan).

***Astrocyte morphology***

The immunofluorescent brain sections stained with anti-GFAP antibodies were imaged with a confocal microscope via the Z-stack protocol every 1 μm between 0 and 30-μm thickness in the hippocampal DG. The Z-stack images were reconstructed into 3D images using the LSM software. The 3D-reconstructed images were subjected to a Sholl analysis with 10-μm concentric circles from the cell body. The number of intersections between astrocyte processes and concentric circles was quantified using Adobe Photoshop CS6 (Adobe Inc., San Jose, CA).

***Astrocyte–neuron interaction***

Astrocyte and post-synaptic markers were imaged with anti-GFAP and anti-PSD-95 antibodies using a confocal-A1 microscope. The images were obtained using a Z-stack system with a 0.5-μm interval between 0 and 30 μm in the hippocampal DG. The stacked image was cut along the X–Z axis using the NIS element software (Nikon Corporation). Astrocyte-neuron interaction were defined by more than 400 nm of PSD-95 labeling within less than 200 nm from the GFAP process. The number of astrocyte-neuron interaction was quantified using Adobe Photoshop CS6.

**Western blotting**

Western blotting protocol was previously described [2]. Briefly, Hippocampi were homogenized with RIPA mix contained protein inhibitor and phosphatase inhibitor. The primary antibodies were applied in the following concentrations: anti-GFAP (rabbit, 1: 5,000), anti-PSD-95 (mouse, 1: 1,000), anti-syanptophysin (mouse, 1:5,000) and anti-GAPDH (rabbit, 1:1,000) for 16 h at 4°C. HRP conjugated secondary antibodies were applied in the following concentrations: anti-rabbit (1:2,000) and anti-mouse (1:2,000) for 1 h at 24°C. The HRP signals were visualized using an enhanced chemiluminescent substrate.

***Statistical analysis***

Data are presented as mean ± standard error of the mean (SEM). A one-way ANOVA followed by Fisher’s LSD post-hoc analysis for comparison with the control group was performed using SPSS (SPSS Inc., Chicago, IL). Statistical significance was set at *p* <0.05.

**References**

1. Choi M, Ahn S, Yang EJ, Kim H, Chong YH, Kim HS. Hippocampus-based contextual memory alters the morphological characteristics of astrocytes in the dentate gyrus. Mol Brain. 2016;9(1):72.
2. Choi M, Kim H, Yang EJ, Kim HS. Inhibition of STAT3 phosphorylation attenuates impairments in learning and memory in 5XFAD mice, an animal model of Alzheimer's disease. J Pharmacol Sci. 2020 Aug;143(4):290-99.
