# Supplementary material for: Disruption of the astrocyte–neuron interaction is responsible for the impairments in learning and memory in 5XFAD mice: an Alzheimer’s disease animal model
Source: Mol Brain. 2021 Jul 10;14:111. doi: 10.1186/s13041-021-00823-5 (PMC8272251; doi:10.1186/s13041-021-00823-5)
Supplement: Supplementary file 2 — Additional file 2. Additional figure for the change of protein expression of GFAP and PSD-95 after Stattic administration in the hippocampus. [file 13041_2021_823_MOESM2_ESM.docx]

**
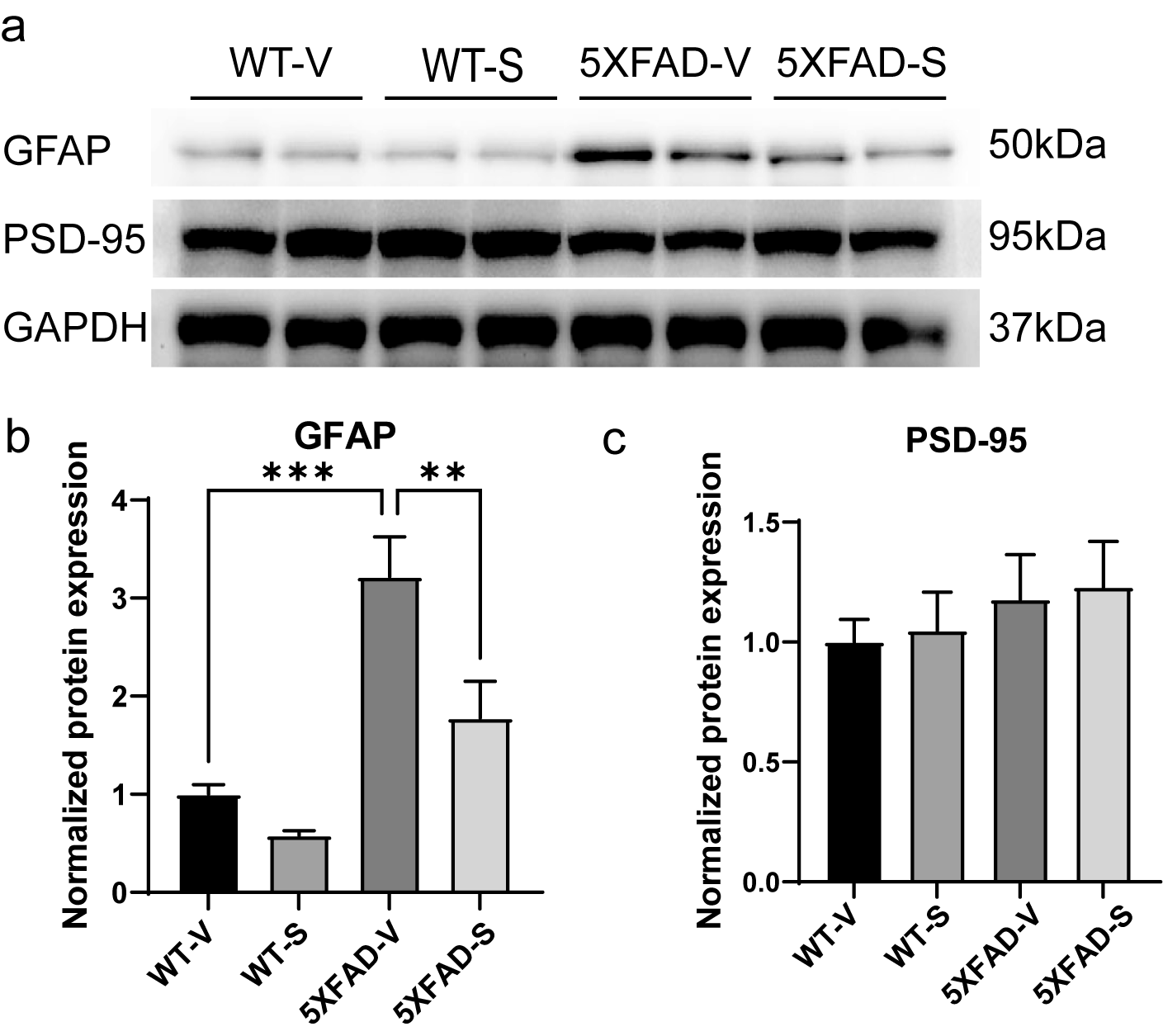
**

**Additional material:**

Figure S1. The change of protein expression of GFAP and PSD-95 after Stattic administration in the hippocampus. **a** Representative immunoblots for GFAP and PSD-95. **b** Densitometric analysis of immunoblots for GFAP and PSD-95 in the vehicle and Stattic treatment groups; WT-V(N=6), WT-S(N=6), 5XFAD-V(N=6), 5XFAD-S(N=6) groups. *p <0.05, **p <0.01, ***p <0.001, one-way ANOVA with Tukey post hoc analysis. Data are presented as mean ± SEM.
